# Preparation of chitosan/retinoic acid @ nanocapsules/TiO_2_ self-cleaning one-dimensional photonic crystals and the study of the visual detection of acute promyelocytic leukemia†

**DOI:** 10.1039/d3ra02224b

**Published:** 2023-06-19

**Authors:** Shuai Li, Zhiqiang Wang, Yanying Chen, Qing Zou, Qianqian Zou, Long Wang, Yanxi Zhu, Lijuan Wang

**Affiliations:** a Qingdao University Qingdao Shandong Province China; b Central Laboratory, Linyi People's Hospital Linyi Shandong Province China zhu-yanxi@163.com wanglj730@163.com; c Laboratory of Hematology, Linyi People's Hospital Linyi Shandong Province China; d Department of Hematology, Linyi People's Hospital Linyi Shandong Province China; e Laboratory Department, Traditional, Chinese Medicine Hospital of Linyi Linyi Shandong Province China 506666569@qq.com; f Key Laboratory of Neurophysiology, Health Commission of Shandong Province Linyi Shandong Province China; g Key Laboratory for Translational Oncolgoy, Xuzhou Medical University Linyi Shandong Province China; h Linyi Key Laboratory of Tumor Biology Linyi Shandong Province China

## Abstract

Sample exposure to air during optical detection leads to the widespread dispersal of microorganisms in the air, posing a health threat to patients and healthcare workers and potentially causing numerous nosocomial infections. In this study, a TiO_2_/CS-nanocapsules-Va visualization sensor was developed by alternatively spin-coating TiO_2_, CS and nanocapsules-Va. The uniformly distributed TiO_2_ can endow the visualization sensor with good photocatalytic performance, and the nanocapsules-Va can bind specifically to the antigen and change its volume. The research results showed that the visualization sensor cannot only detect acute promyelocytic leukemia conveniently, quickly and accurately, but also kill bacteria, decompose organic residues in blood samples under the influence of sunlight, and have an extensive application prospect in substance detection and disease diagnosis.

## Abstract 2

The visualization sensor cannot only detect acute promyelocytic leukemia conveniently, quickly and accurately, but also kill bacteria and decompose organic residues in blood samples under the influence of sunlight.

## Graphical abstract

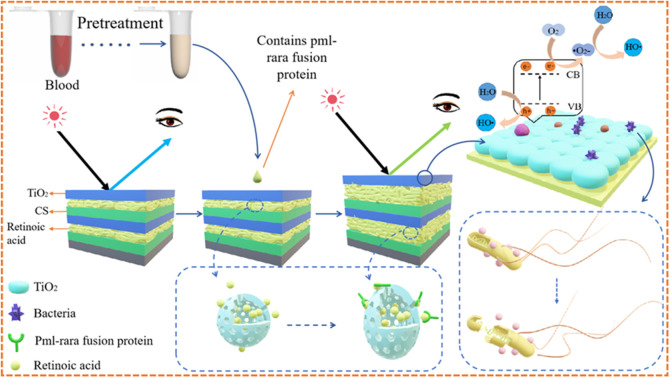

## 1. Introduction

Acute promyelocytic leukemia (APL) is a subtype of acute myeloid leukemia^1,2^ with an extremely high risk of premature death.^3^ Late diagnosis and treatment of APL leads to its rapid deterioration and causes disseminated intravascular coagulation (DIC) within a short period,^4,5^ eventually leading to death. Understanding how to quickly and accurately diagnose a disease in its early stages is crucial. Currently, the diagnosis of APL mainly relies on flow cytometry and fluorescence *in situ* hybridization (FISH), among other tests. However, these tests are associated with constraints, including high cost, long operation time, and advanced technical requirements, thus limiting their application.

Using a one-dimensional photonic visual sensor for APL diagnosis has many advantages, including: (1) visualization; the visual sensor can show the changes in the internal structures through colour changes. Moreover, by comparing the differences in the colours of the sensor before and after testing, the disease can be diagnosed.^6^ (2) Fast; visual sensors can perform a test and diagnose patients at any time. The detection takes between 10–20 min. (3) Accurate; the chelation of specific targeting substances with photon band gap can fully ensure the sensitivity and accuracy of detection due to the unique photonic band gap structure inside the sensor. (4) The preparation and operation are simple; the production of a visual sensor only requires a single instrument, while the production is simple and easy to learn. (5) Economical; the raw materials for producing visual sensors are cheap and commonly used reagents in the laboratory, including glacial acetic acid, ethanol, chitosan, silicon wafer, tetrabutyl titanate, *etc.* Most of the raw materials are common reagents in the laboratory. (6) Strong inclusivity; the one-dimensional photonic crystal imaging sensor has excellent inclusivity and can practically be assembled with most substances. In addition to various drugs, it can also be embedded with antigens, antibodies, glycoproteins, and other biologically active substances. Different diseases can be diagnosed by altering the implanted materials.^7^

However, during optical detection, the test samples will inevitably be exposed to the air, posing certain challenges. If the sample includes infectious pathogenic bacteria, they will spread in the air during relevant testing, seriously endangering the health of the operator. Moreover, improper handling of samples is among the leading causes of nosocomial infections. Multiple drug-resistant bacteria such as MASA (methicillin-resistant *Staphylococcus aureus*), VRE (vancomycin-resistant *enterococcus*), and CRE (penicillium carbon alkene resistant *E. coli*), among others, often appear in the hospital from occupational exposure to infected blood samples from patients. These resistant bacteria spread rapidly and are also challenging to treat.^8^ Additionally, proper disposal of residual waste after testing is a major concern. Improper disposal pollutes the environment while burning leads to wastage of resources and smoke pollution.

Design and development of a visual sensor with properties such as self-cleaning, antibacterial, and the potential to clear associated residual waste after the test will effectively protect the health workers, reduce nosocomial infections, protect the environment, and reduce environmental pollution. TiO_2_ is an excellent photocatalytic material and has been widely used in various self-cleaning designs.^9,10^ Photoexcitation of TiO_2_ produces superoxide anion and hydroxyl radicals,^11^ which have strong oxidation capacity and interacts with bacteria and viruses, decomposing them by oxidation, suggesting potential utilization in disinfection and sterilization,^12,13^ TiO_2_ has numerous advantages, compared with conventional antibiotics, including a broad antibacterial spectrum, high sterilizing rate, high safety rating, ease of storage, and high bacterial tolerance.^14^ In addition, the superoxide anions and hydroxyl radicals combine with different organic wastes and eliminate them by oxidative breakdown. Therefore, this experiment aimed to incorporate TiO_2_ into the visual sensor design. The TiO_2_ visual sensor maintained the sensitivity of a photonic crystal while inheriting the photocatalytic and antibacterial properties of TiO_2_.^15^ It cannot only realize the rapid diagnosis of diseases, but also kill pathogenic microorganisms in samples and thoroughly decompose residual waste, prevent nosocomial infection and avoid environmental pollution.

## 2. Experimental

### 2.1. Experimental reagent

Retinoic acid, Tetrabutyl titanate, and Rhodamine B (RB) were purchased from Shanghai MackLin Biochemical Co., Ltd; absolute ethanol (analytical grade), and glacial acetic acid (analytical grade), 98% concentrated sulfuric acid (analytical grade), 30% hydrogen peroxide (analytical grade) and chitosan were purchased from Sinopharm Chemical Reagent Co., Ltd; silica was purchased from XFNANO company; polyacrylic acid (PAA) and polyethyleneimine (bPEI) were purchased from Sigma-Aldrich; water was made in the laboratory.

### 2.2. Preparation of a visual sensor

TiO_2_ gel: 4 mL tetrabutyl titanate and 4 mL glacial acetic acid were slowly added into 16 mL absolute ethanol and mixed with a magnetic stirrer for 5 h at room temperature to prepare light yellow TiO_2_ solution. 1 mL of TiO_2_ solution was diluted by adding 2 mL absolute ethanol. Glacial acetic acid was used to lower the pH of the gel, and 2 mL chitosan (CS) solution with a concentration of 4 mg mL^−1^ was added to the TiO_2_ solution and mixed well to prepare the TiO_2_ gel.^16^

Retinoic acid nanocapsules (nanocapsules-Va): pure water was used as a solvent, with a 4 mg mL^−1^ of polyacrylic acid (PAA) and polyethylene imine (bPEI) solution. 300 nm SiO_2_ particles were washed in pure water and centrifuged to obtain a clean SiO_2_ core. The SiO_2_ core was immersed in 4 mg mL^−1^ PAA solution for 10 min, centrifuged at 1500 rpm for 5–10 min, then immersed in bPEI for 5–10 min and centrifuged at 1500 rpm for 5–10 min. The above operation was repeated twice to obtain SiO_2_-(PAA/bPEI)_2_. After washing, SiO_2_-(PAA/bPEI)_2_ was immersed in a 4% HF acid solution for 30 min and centrifuged at 800 rpm for 30 min to separate the sediments. Hollow nanocapsules were obtained by washing with water and centrifugation 2–3 times. The aqueous solution of the nanocapsule was then frozen in the refrigerator for 1–2 h. After the two hours, the frozen nanocapsules were quickly placed in the freeze dryer for 15–24 h. Retinoic acid was dissolved in absolute ethanol to make a retinoic acid ethanol solution. Completely dry nanocapsules were dissolved in the retinoic acid ethanol solution and kept in the refrigerator for 15–24 h to make nanocapsules-Va.

**Fig. 1:**
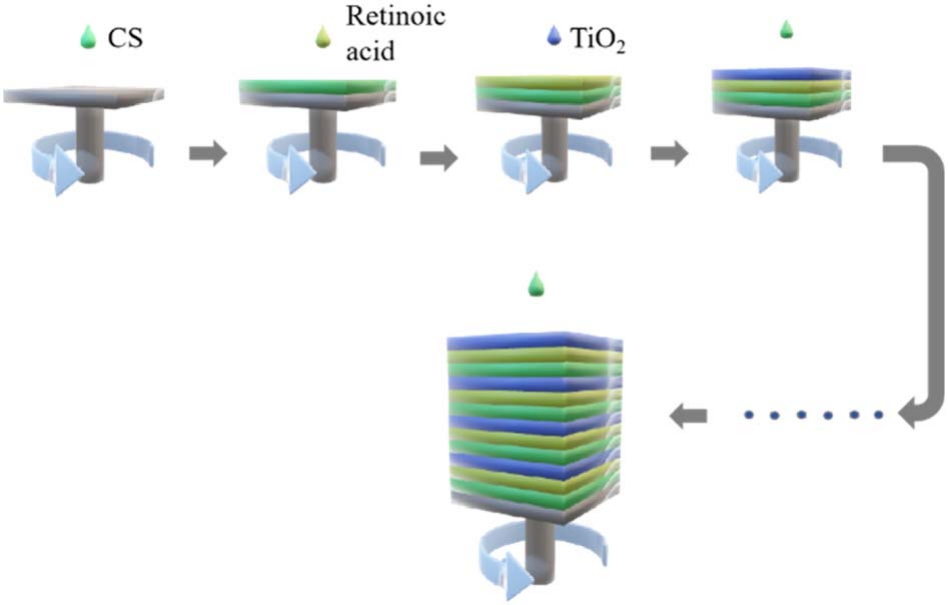
Schematic diagram of one-dimensional photonic crystal preparation process.

Clean the surface dust of the silicon with pure water and immerse it in piranha solution for 24 h, then wash the surface of the silicon wafer with absolute ethanol and blow dry with nitrogen. The prepared TiO_2_ gel was uniformly spin-coated on the surface of the silicon wafer by a spin coater (Fig. 1). The rotation speed was controlled at 2000–4000 rpm, and the gel was dried at 30 °C for 15 min in a vacuum drying oven. In the second layer, nanocapsule-Va was uniformly coated on the surface of the silicon wafer at a rotational speed of 2000–4000 rpm, and dried in a vacuum drying oven for 15 min. The third layer was spin on the surface of the silicon wafer with a layer of water-soluble CS solution at 2000–4000 rpm and dried in the vacuum drying oven for 15 min. Visual sensing sensor were developed by alternatively spin-coating for 4–7 cycles as previously described.

### 2.3. Visual detection of APL by the visual sensor

1 mL of blood from APL patients was centrifuged for 5 min at 800 rpm. After centrifugation, the supernatant and white blood cells were collected, while the lower section, composed of red blood cells, was discarded. Pure water was added to the retained plasma and white blood cells to dissolve the white blood cells, and fully release the cellular components. The collected mixed liquid droplets of plasma leukocytes were then added to the surface of a one-dimensional photonic crystal, and allowed to stay for 15 min before observation for the colour change. An optical fibre spectrometer was used to detect the spectral changes.

### 2.4. Visual sensor degradation of organic matter experiment

A batch of visual sensors with the same parameters and raw materials was prepared and arranged in a tray. Add 4 mg L^−1^ RB solution 200 μL on each surface of the visual sensor. A UV lamp was used to continuously irradiate at room temperature, and the RB liquid on the sensor surface was sampled once every 30 min. Irradiation was halted when the liquid on the sensor surface turned colourless. The colourless solution was collected for storage. A multifunctional microplate reader (molecular devices) was used to measure the absorption peaks of all samples, collated and plotted.

### 2.5. The antibacterial experiment of visual sensor

In addition to promoting the decomposition of organic matter, TiO_2_ has excellent antibacterial properties, which can prevent and kill common pathogenic bacteria. The following experiments were conducted to explore the antibacterial performance of the visual sensor containing TiO_2_. The bacterial solution containing a high concentration of *E. coli* was diluted 10 times, 100 times, 1000 times, 2000 times, 5000 times, and 10 000 times for bacterial culture. 2 mL of the diluted bacterial solution was added to the medium, shaken until evenly distributed over the surface of the medium, and the excess bacterial solution was discarded. The culture medium inoculated with a bacterial solution was incubated at 37 °C for 24 h to initiate colony formation and growth. The bacterial solutions with the best growth state were selected for subsequent antibacterial experiments. The antibacterial experiment was conducted using a bacterial solution diluted 10 000 times. The CS solution was assembled on the surface of the silicon wafer using the spin coating method, resulting in an even layer of CS film covering the entire surface of the silicon wafer. The visual sensor containing TiO_2_ (TiO_2_–Si), pure silicon wafer (Si), and silicon wafer covered with chitosan film (CS–Si) were irradiated by UV light for > 2 h to kill the remaining bacteria on the surface completely. 100 μL pure water was added to the surface of TiO_2_–Si, Si, and CS–Si, respectively, and irradiation continued for 4 h. Water was occasionally added to the surfaces of the three silicon wafers during the irradiation process to ensure they were always moist. The UV light was turned off after 4 h of irradiation, and 20 μL of the bacterial solution was dropped on the surface of the three silicon wafers and left for 1 h at room temperature. The liquid on the surface of TiO_2_–Si, Si, and CS–Si was absorbed and stored after 1 h. The recovered liquid was diluted to 2 mL with water for culturing convenience and labelled as “Bacteria–TiO_2_”, “Bacteria–Si”, and “Bacteria–CS”, respectively. 20 μL of the original bacterial solution was diluted to 2 mL with water and labelled as “Bacteria–C”. The four bacterial samples were incubated for 24 h at 37 °C. The growth status of the colonies was evaluated and recorded using a chemiluminescence imager (protein simple).

## 3. Results and discussion

### 3.1. Characterization of visual sensors

**Fig. 2:**
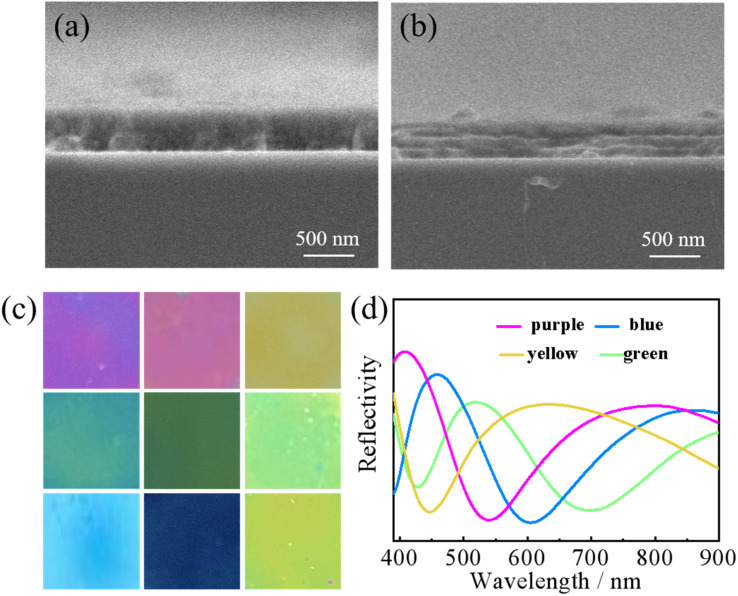
(a) SEM cross section of the visualization sensor without nanocapsules. (b) SEM cross section of the sensor visualized with nanocapsules. (c) Visual sensors in different colours. (d) Reflection spectra of visualization sensors with different colours.

The spin coating approach was used in this work to create a one-dimensional photonic crystal imaging sensor. The thickness of each layer was adjusted by changing the rotation speed of the spin coating instrument. We used different speed and control the number of layers of the film to fabricate the one-dimensional photonic crystal visualization sensors: 2000 rpm–4000 rpm. The colour change of the visual sensor is shown in Fig. 2(c) and can be displayed in different colours, including yellow, yellow-green, green, blue-green, blue, purple, and purple-red, among others. The spectrum coverage is shown in Fig. 2(d), from 400 nm to 700 nm, covering almost all the wavelength ranges of visible light. The position of photonic band gap is directly determined by adjusting the rotational speed. The position of the photonic band gap inside the sensor changes, resulting in a change in colour for the one-dimensional photonic crystal visualization sensor.^17^ Variety media types are periodically arranged in layers causing predictable fluctuations in the refractive indices.^7^ Because of the periodic superposition of different media, the internal refractive index of the crystal film presents periodic changes. When the change of refractive index matches a certain wavelength in the optical band, it will block the propagation of light waves in the crystal from all directions, which is the photonic band gap.^18^ The rotation speed during the fabrication of photonic crystal will directly affect the thickness of the photonic crystal film, and the change of thickness will change the periodic fluctuation trend of refractive index, resulting in the shift of photonic band gap.^19,20^

At the microscopic level, the 500 nm scanning electron microscope (SEM) section of the visualization sensor is shown in Fig. 2(a) and (b). The sensor shown in Fig. 2(a) was made without the addition of nanocapsules, and the layer to layer arrangement in the section diagram is very tight, making it difficult to see the layered structure. Fig. 2(b) shows the SEM cross section of the visual sensor after the addition of nanocapsules. The cross section shows obvious layered structures, and scattered nanocapsules can be seen inside the sensor. In the sensor shown in Fig. 2(a), the sensor is solely made of TiO_2_ gel and CS solution alternately coated. TiO_2_ gel contains a small amount of CS, which promotes good compatibility between the two layers. In addition, the high speed makes the TiO_2_ gel and CS film thin, while the tight-fitting makes the sensor stratification in Fig. 2(a) not obvious. In Fig. 2(b), a layer of nanocapsule coated with retinoic acid is added between the TiO_2_ and CS layers of the sensor, making the sensor stratification more obvious. The addition of nanocapsules, and making the sensor more stratified is essential for protecting the internal retinoic acid drug molecules. Retinoic acid has poor stability and is prone to denaturation when exposed to high temperatures, bright light, and extreme pH, among other conditions. To avoid the denaturation of retinoic acid, we encase drug molecules in a nanocapsule shell.^21^ Under the protection of the nanocapsules shell, retinoic acid maintains its stability. During sample detection, the drug molecules of retinoic acid in the nanocapsule and attached to the surface of the nanocapsules shell will recognize and bind the targeted protein, leading to the deformation of the nanocapsules. The structure and volume of the entire nanocapsules layer will also change to enhance the detection of drug molecules. The cleaning task is completed by the TiO_2_ gel layer. Through the photocatalytic effect, TiO_2_ molecules eliminate pathogenic microbes and decompose residual wastes to achieve self-cleaning. In addition, under the protection of nanocapsules, even after exposure to light retinoic acid will not be seriously damaged, and can still complete the detection task normally. In conclusion, compared with Fig. 2(a), the hierarchical structure of Fig. 2(b) has more advantages. It protects the internal retinoic acid from being destroyed, allowing the completion of the detection task while ensuring smooth self-cleaning of the samples.

### 3.2. Visual detection of APL

**Fig. 3:**
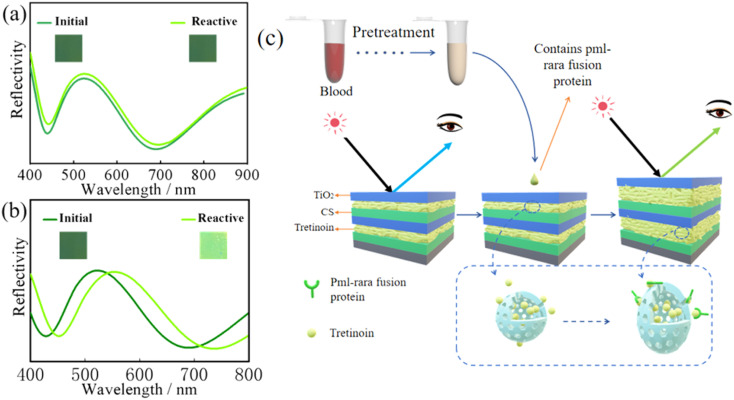
(a) Visual sensors detect colour changes in normal human blood samples before and after, (b) visual sensors detect colour changes in APL blood samples before and after, (c) visual sensor detection mechanism diagram.

Pre-treated blood samples from normal people and APL patients were dropped onto the surface of the visual sensor and allowed to stay for 15–30 min. The colour changes in the sensor were then observed. The optical fibre spectrometer was used to measure the reflection spectrum, as shown in Fig. 3(a) and (b). The initial colour of the sensor was dark green, and the initial curve represented its reflection spectrum. The peak was located near 520 nm. There was no obvious change in the visual sensor when normal blood samples were added, and the reflection spectrum before and after the sensor detection was around 520 nm without significant displacement, indicating that the blood of normal people could not make the sensor react (Fig. 3(a)). But the visual sensor that drops APL patients' blood samples turns light green and its peak had shifted to the right, appearing near 550 nm (Fig. 3(b)). It also reflects that the visual sensor has high sensitivity and specificity for APL diagnosis.

The reaction mechanism is shown in Fig. 3(c). Due to a gene mutation, APL patients have a unique fusion gene referred to as PML–RARA.^22,23^ The PML–RARA fusion gene will guide cells to produce a special protein called PML–RARA fusion protein, which is also responsible for blood coagulation disorder in APL patients. Due to their high specificity, the fusion gene and fusion protein are often used as markers in APL diagnosis.^24,25^ The RARA terminal of the PML–RARA fusion protein is a retinoic acid receptor,^26,27^ which recognizes and binds retinoic acid.^28^ In the current study, we embedded retinoic acid into the sensor despite the associated properties, such as ease of decomposition under adverse conditions, including extreme light, high temperatures, and extreme PH. To overcome these challenges, we used nanocapsules to encase retinoic acid to prevent its exposure to external adverse conditions.^29,30^ After dropping the blood sample on the surface of the visual sensor, nano-encapsulation internal retinoic acid was detected. Based on the PML–RARA fusion protein in the blood sample, the combination of internal materials induces changes in the thickness of the layer structure, which leads to a change in the colour of the sensor surface and realization of APL visual diagnosis.

It is easier, quicker, and more practical to use visual detection than the conventional methods because there is no need to operate complicated instruments; instead, test results can be obtained by placing the processed blood sample on the surface of the ready visual sensor and waiting 10 to 20 min. In addition, the production of the visual sensor is simple, and the production cost is low. Most of the raw materials needed are common reagents in the laboratory. When developing a sensor, the selected medium material can be coated on the surface of the silicon wafer periodically in the established order. Moreover, the photonic band gap of the visual sensor is sensitive to changes in its internal structure and volume, improving the sensitivity of the visual sensor. The retinoic acid inside the sensor can accurately identify and bind to PML–RARA fusion protein, which ensures the specificity of the sensor for APL. Consequently, as a new detection device, the visual sensor developed in this experiment has the potential for accurate diagnosis of diseases and has numerous such as simple operation, convenient use, fast, and low cost, which have excellent development prospects.

### 3.3. Visual sensor photocatalytic decomposition of organic matter

**Fig. 4:**
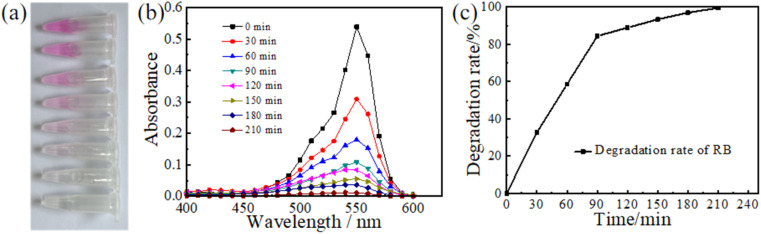
(a) Colour change of RB in the process of catalytic decomposition of the sensor surface under UV irradiation. (b) Change of absorption peak of RB solution in different time periods. (c) Decomposition rate curve of RB.

The photocatalysis capacity of TiO_2_ has been widely applied in various self-cleaning surfaces since its discovery by Fujishima and his colleagues.^31^ In presence of sunlight and UV light, TiO_2_ has the potential to decompose most organic compounds.^32,33^ Therefore, the TiO_2_ gel-based visual sensor also possesses excellent photocatalysis. To study the degradation ability of TiO_2_ visual sensor on organic matters, we took RB as the experimental object to conduct exploratory experiments. RB solution was dropped on the surface of the TiO_2_ visualization sensor and irradiated with UV light. Samples were taken every 30 min to observe the decomposition of RB. As shown in Fig. 4(a), RB undergoes decomposition under UV irradiation, gradually losing its initial dark purple colour and eventually turning colourless. The decomposition takes about 4 h. The residual RB in the solution can then be determined by measuring the spectral absorption peak for each sample. The peak changes are shown in Fig. 4(b). The optimum absorption peak was observed at 0 min, which corresponds to the initial solution and the maximum concentration of RB in the sample at that time. At 210 min, the absorption peak values were lowest indicating that the concentration of RB was almost negligible at that point. As a result, we assumed that RB had been completely decomposed at 210 min. Maximum values of the absorption peaks were obtained and used to draw graphs and calculate the decomposition rate of RB. As shown in Fig. 4(c), the decomposition rate of RB was fast before 90 min, close to 0.864% per min. About 77.76% of RB was decomposed at this stage. At 90 min, a turning point appeared, the slope of the decomposition curve shifted, and the decomposition rate of RB decreased. At 210 min, the degradation efficiency of RB reached 99.5%. Our results show that the photocatalytic performance of a one-dimensional photonic crystal containing titanium dioxide is exemplary, and the total decomposition of RB can be achieved in a short time. Most organic compounds can be photo catalytically decomposed by RB and a visual sensor composed of TiO_2_.^34^ The structural properties of TiO_2_ have been associated with its excellent decomposition capacity. The atomic or molecular orbital of TiO_2_ has an empty region between the valence band and the conduction band. The exposure of TiO_2_ to sunlight, especially UV radiation leads to an increase in the electron energy. When the electron energy reaches the band gap energy, the electrons are excited from the valence band to the conduction band, generating an electron–hole pair.^35^ Under the effect of an electric field, the electrons and holes are separated, and the electrons migrate to the particle surface. It forms a superoxide anion (˙O_2−_) with the oxygen adsorbed on the surface of TiO_2_. The anion free radical of ˙O_2−_ reacts with most organic compounds by oxidation to generate CO_2_ and H_2_O,^36,37^ while the hole oxidizes OH and H_2_O adsorbed on the surface of TiO_2_ to ˙OH.^38,39^ At the same time, ˙O_2−_ reacts with H_2_O oxidizing it to ˙OH. ˙OH has a strong oxidizing ability, which can attack organic compounds and extract H atoms to generate new free radicals, stimulate chain reactions and lead to the decomposition of organic matter.^33^ TiO_2_ has a high catalytic activity and good chemical stability. As a result, the visual sensor containing TiO_2_ can effectively remove the organic residues after the experiment. Compared with traditional waste treatment methods, the catalytic degradation of the visual sensor does not require the addition of chemicals, or the high-temperature and high-pressure treatment. The use of sunlight or ultraviolet lamps can realize the decomposition of waste, not only safe and convenient, but also energy saving. Our results showed that the visual sensor can decompose and remove most of the organics in a short time, and has a high degradation efficiency. In addition, the final products of the catalytic decomposition of organic compounds by visual sensors are H_2_O and CO_2_,^40^ which do not emit pollutants. As a result, the release of the decomposition by products into the natural environment will not be detrimental suggesting the use of visual sensors for diagnosis thus promoting green environmental protection.^41^

### 3.4. Antibacterial properties of the visual sensor

**Fig. 5:**
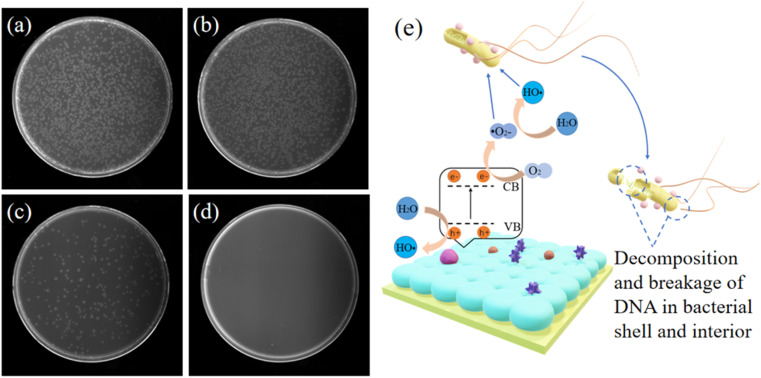
(a) The distribution of *Escherichia coli* colonies on the surface of pure silicon wafer after 24 h culture; (b) colony status of original bacterial solution after 24 h of culture; (c) the distribution of *Escherichia coli* colonies on the surface of CS membrane after 24 h culture; (d) The distribution of *Escherichia coli* colonies on the surface of visual sensor containing titanium dioxide after 24 h culture; (e) schematic diagram of antibacterial mechanism of visual sensor.

In addition to the photocatalytic decomposition of organic compounds, TiO_2_ has excellent antibacterial properties. Therefore, TiO_2_ visual sensors have excellent antibacterial properties.^42^ TiO_2_ has a broad antibacterial spectrum and can eliminate the majority of common bacteria, viruses, mycoplasmas, and other pathogenic microorganisms.^43^ In this study, *E. coli* was used to test the antibacterial performance of the TiO_2_ visual sensor. The experimental results are shown in Fig. 5(a)–(d). The results showed that a large number of colonies were cultured in the original bacterial solution and the bacterial solution on the surface of the silicon chip. The number of coliform colonies cultured in the bacterial solution on the surface of the chitosan membrane was significantly low, while no colonies were cultured in the bacterial solution treated with TiO_2_ visual sensor. Our results indicated that although CS is bactericidal,^44,45^ its potency is not enough to completely inhibit bacterial growth. On the other hand, the visual sensor containing TiO_2_ eliminated bacteria.

TiO_2_ sterilizes primarily in two ways, as highlighted in Fig. 5(e). Firstly, after exposing TiO_2_ inside a one-dimensional photonic crystal to UV light, the excited holes directly act on each part of the bacteria, directly oxidizing and decomposing the cell wall, cell membrane, cytoplasm, nucleic acid, *etc.* killing the bacteria. The second way is similar to the photocatalytic decomposition of organic matter. TiO_2_ generates ˙O_2−_ and ˙OH after UV radiation, which have super oxidizing properties and can react with most organic compounds, bacteria, and other pathogenic microorganisms.^46,47^ The radicals oxidize and decompose bacteria and viruses into H_2_O and CO_2_ for sterilization and disinfection. Therefore, using the visual sensor developed in this study will decompose and eliminate infectious pathogenic microbes in the test sample during diagnosis. The diagnosis method proposed in this study using the visual sensor effectively protects the healthcare staff and prevents nosocomial infections caused by the large-scale transmission of pathogenic microbes. This is critical to reduce nosocomial infections, safeguard medical professionals and protect hospitalized patients from developing secondary infections.

## 4. Conclusions

The visual sensor was designed and developed in this paper, sensitively detected the special fusion protein in blood samples by its photonic band gap structure, and achieved rapid and accurate diagnosis of APL. Compared with traditional detection methods, it had strong specificity, high sensitivity, convenient production, and low production cost. In addition, the visual sensor also had an excellent self-cleaning ability. Its internal TiO_2_ molecules catalysed H_2_O and O_2_ to generate strongly oxidizing ˙O_2−_ and ˙OH under the influence of UV light, and oxidized and decomposed most organic substances. It was observed that the sensor could kill pathogenic microorganisms, avoid their spread in samples, reduce nosocomial infections, and decompose and remove residual waste, degrading them into H_2_O and CO_2_, thus reducing the environmental pollution. The degradation was efficient, and the degradation products were clean and pollution-free, consistent with the concept of green environmental protection, and has an extensive application prospect in substance detection and disease diagnosis.

## Ethical statement

Informed consents were obtained from human participants of this study.

## Author contributions

SL, ZQW: methodology; SL, ZQW, QQZ: formal analysis, investigation and resources; SL: data curation, and writing – original draft preparation; LJW, YXZ, QQZ: writing – review & editing, visualization; YYC, QZ, LW, YXZ, LJW: supervision, project administration. LJW: funding acquisition. All authors have read and agreed to the published version.

## Conflicts of interest

There are no conflicts to declare.

## Supplementary Material

RA-013-D3RA02224B-s001
