# Supplementary figures and images for: Preparation of chitosan/retinoic acid @ nanocapsules/TiO2 self-cleaning one-dimensional photonic crystals and the study of the visual detection of acute promyelocytic leukemia
Source: RSC Adv. 2023 Jun 19;13(27):18363–70. doi: 10.1039/d3ra02224b (PMC10277903; doi:10.1039/d3ra02224b)

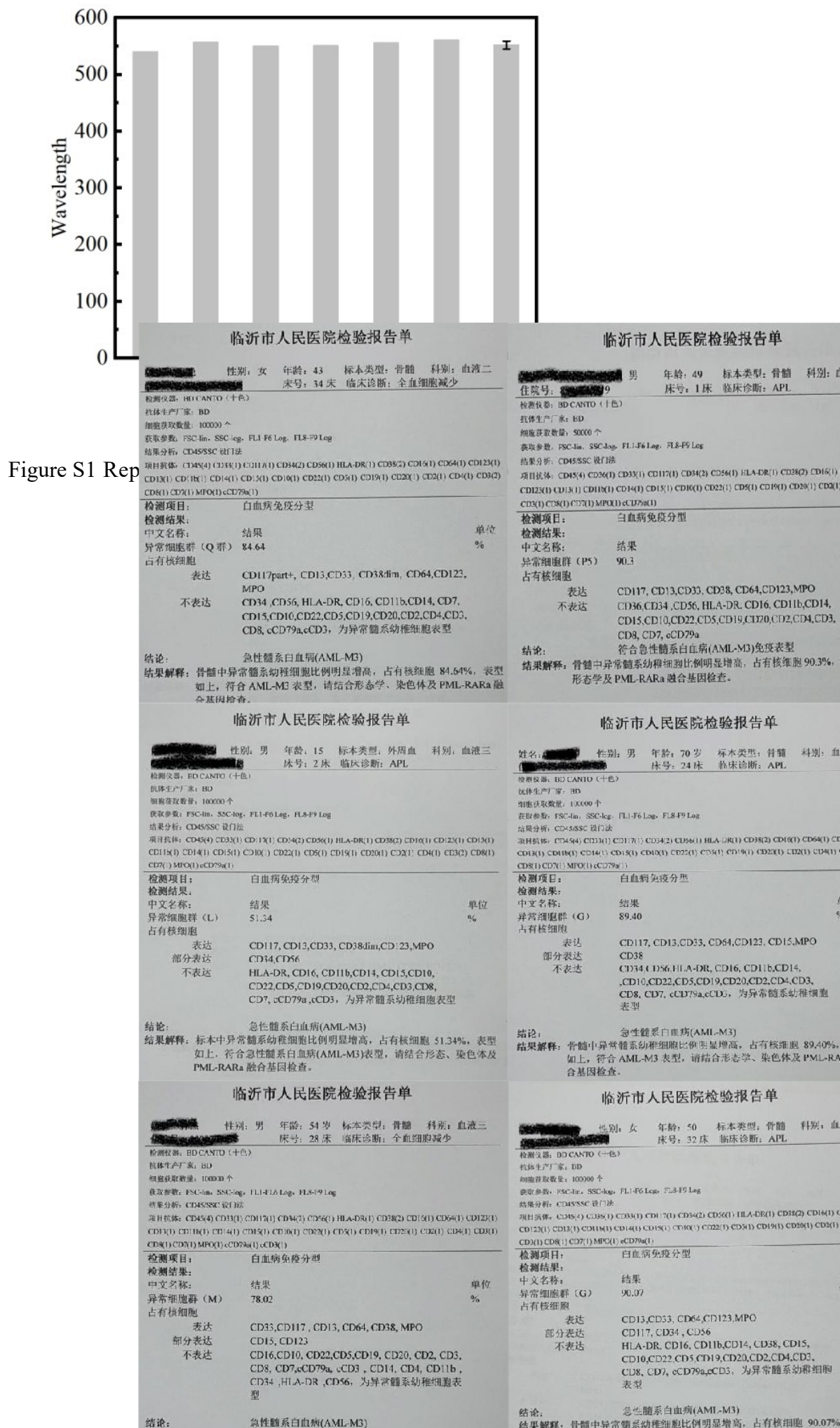

FigureS2 Provide clinical diagnosis reports of patients with blood samples.

Supplement: RA-013-D3RA02224B-s001 [file RA-013-D3RA02224B-s001.pdf]
